# A Literature Review of Lateral Epicondylitis: Diagnosis, Risk Factors, Management and Treatment

**DOI:** 10.3390/life16071043

**Published:** 2026-06-23

**Authors:** Emilia Biedroń, Maciej Pitra, Jakub Chmura, Mikołaj Zieliński, Grzegorz Fibiger, Dawid Plutecki, Andrzej Dubrowski, Kamil Możdżeń, Jerzy A. Walocha, Wojciech Fibiger, Tomasz Kozioł

**Affiliations:** 1 Faculty of Medicine and Health Sciences, University of Applied Sciences in Tarnow, 33-100 Tarnów, Poland; emilia.biedron44@gmail.com (E.B.); maciej.pitra@interia.pl (M.P.); jakub_chmura@interia.pl (J.C.); mikolaj.z5@onet.pl (M.Z.); 2 International Evidence-Based Anatomy Working Group, 31-034 Kraków, Poland; 3 Department of Anatomy, Jagiellonian University Medical College, 31-034 Kraków, Poland; dawid.plutecki@uj.edu.pl (D.P.); tomasz1.koziol@uj.edu.pl (T.K.); 4 ARTROMED Orthopedic and Rehabilitation Center, 30-059 Kraków, Poland; fibigerw@mp.pl; 5 Department of Orthopedics and Traumatology, Jagiellonian University Medical College, 30-688 Kraków, Poland; 6 Department of Clinical Physiotherapy, Institute of Health, University of Applied Sciences in Nowy Targ, 34-400 Nowy Targ, Poland

**Keywords:** lateral epicondylitis, lateral elbow tendinopathy, tennis elbow, conservative treatment, rehabilitation

## Abstract

Lateral epicondylitis (LE), commonly referred to as tennis elbow, remains a frequent cause of lateral elbow pain, yet its optimal management and risk profile are still debated. Therefore, this review aimed to summarize current evidence on its definition, diagnosis, and treatment while addressing common misconceptions. A non-systematic review of major medical databases, including PubMed, Cochrane Library, and Google Scholar, was conducted using predefined inclusion criteria to identify relevant review articles. The analyzed literature highlights that LE is primarily diagnosed clinically and managed through a spectrum of conservative and interventional approaches. Evidence suggests that structured physiotherapy and load modification remain the cornerstones of treatment, while modalities such as platelet-rich plasma and autologous blood injections may offer longer-term benefits compared with corticosteroids, which are effective mainly for short-term symptom relief. In contrast, interventions such as acupuncture and shock wave therapy show limited or inconsistent efficacy. Identified risk factors include female sex, smoking history, repetitive or forceful manual work, and higher cardiovascular risk burden. Overall, conservative management should be the first-line approach, with biologic therapies considered in refractory cases and surgery reserved as a last option; however, further high-quality randomized controlled trials are required to establish optimal treatment algorithms and clarify long-term outcomes.

## 1. Introduction

Lateral epicondylitis (LE) is a tendinopathy of the forearm extensor muscles that attach to the lateral epicondyle of the humerus, such as extensor carpi radialis brevis, extensor digitorum, extensor digiti minimi, extensor carpi ulnaris and anconeus. Historically, “lateral epicondylitis” has been used to describe the pain originating from the common extensor tendon insertion at the lateral epicondyle. However, increasing histopathological evidence suggests that the condition is predominantly degenerative rather than inflammatory, with the presence of angiofibroblastic hyperplasia, collagen disorganization, fibroblast proliferation, or neovascularization [1]. Therefore, the term “lateral elbow tendinopathy” is considered more pathophysiologically [2]. Although, “lateral epicondylitis” remains the most widely recognized and commonly used clinical term in both the literature and clinical practice. The term “tennis elbow” has persisted since its initial description by Morris as “lawn tennis arm” in 1883. Interestingly, fewer than 10% of patients with this condition are actually tennis players [3]. The main reasons for LE are degenerative changes caused by long-lasting overuse of the forearm muscles previously described. Histologically, it presents signs of tendon degeneration, such as the presence of fibroblasts, vascular hyperalgesia and disorganized collagen [4]. Repetitive physical strain may cause microdamage within the tendon origin, leading to degeneration and localized pain. Tendons collagen fibers are damaged causing severe pain on the lateral side of the elbow joint. This pain can be more intense and agonizing while grabbing things, clenching fists, pronating the forearm or straightening the wrist. These activities lead to microtearing most commonly at the origin of the extensor carpi radialis brevis (ECRB) tendon. Moreover, there can be swelling visible on the outer side of the elbow. The pain is mostly localized in the epicondyle but it can radiate to the wrist. It is usually triggered by exerting pressure on the epicondyle and stretching of the epicondylar muscles. LE has an approximate rate of 40% and a prevalence of 1–3% of the general population, being most common in the age range of 35–54 years [4]. LE is a public health issue due to its high frequency in manual workers, among whom 10.5% may experience lateral elbow pain and 2.4% have a confirmed diagnosis of LE [5]. The risk of this tendinopathy is higher in heavy manual laborers and in workers whose job requires repetitive motions or motor skills.

However, despite the prevalence of LE and the growing number of available diagnostic and therapeutic approaches, the current literature remains heterogeneous. In addition, regenerative therapies and imaging techniques have further expanded the management options for this condition, creating the need for an updated and clinical synthesis of the available evidence. Therefore, the purpose of this study is to summarize and critically discuss the current literature regarding this well-recognized yet still unresolved clinical problem, including diagnosis, risk factors, management and treatment strategies for LE.

## 2. Materials and Methods

### Inclusion and Exclusion Criteria

To perform this literature review, a non-systematic search (from May 2025 to February 2026) was conducted in which all review articles published within the scope of the inclusion criteria were looked for. Online medical databases such as PubMed, Cochrane Library, Embase, Scopus, Web of Science Core Collection and Google Scholar were searched through. The search was conducted without a predefined date restriction for publication year. The following keywords and their combinations were used: “lateral epicondylitis,” “tennis elbow,” “lateral elbow tendinopathy,” “common extensor tendon,” “diagnosis,” “risk factors,” “treatment,” “management,” “injection therapy,” “physiotherapy,” and “surgery.” The inclusion criteria were set as follows: randomized controlled trials, prospective and retrospective cohort studies, observational studies, systematic reviews, and meta-analyses published in English were included if they contained original or clinically relevant data related to LE. Preference was given to higher levels of evidence, especially randomized controlled trials, systematic reviews, and meta-analyses when these were available. When evidence conflicted, more importance was placed on studies with larger patient populations, stricter methodologies, longer follow-up periods, and better quality in evidence synthesis. The exclusion criteria were set as follows: case reports, animal studies, conference abstracts, editorials, letters to the editor, expert opinions, and narrative reviews that lacked primary data. Studies not directly related to LE or focusing on unrelated elbow conditions were also excluded. The study selection process was based on title and abstract screening followed by full-text evaluation of potentially relevant articles. Reference lists of selected studies were additionally screened to identify further relevant publications. The overarching goal was to map the spectrum of currently used diagnostic and therapeutic approaches rather than to perform a formal comparative effectiveness analysis.

## 3. Results

### 3.1. Injuries

There are two primary types of injury associated with tennis elbow: chronic and acute. Chronic LE develops gradually due to repetitive overload, particularly of the extensor carpi radialis brevis tendon leading to microtears, collagen disorganization, fibroblast proliferation, and neovascularization, a pattern characteristic of tendinopathy rather than true inflammation. In contrast, acute LE can result from sudden overload such as lifting a heavy object with a straight elbow, which may cause microperforations or partial ruptures of the tendon fibers [6].

An often-overlooked component is nerve compression, especially posterior interosseous nerve (PIN) syndrome—which can mimic or exacerbate LE symptoms. Studies report that approximately 30–40% of patients with chronic lateral elbow pain exhibit both tendinopathy and radial nerve compression [7].

In cases of persistent or treatment-resistant symptoms, it is advisable to perform diagnostic imaging (ultrasound, MRI) and electrophysiological studies to rule out nerve involvement or other pathologies (e.g., elbow instability). Such comprehensive diagnostics can improve treatment planning and outcomes.

### 3.2. Clinical Manifestations and Diagnosis

Pain in the lateral elbow suggests that it is a pain radiating distally along the extensor muscle mass, exacerbated by wrist and finger extension against resistance [5,8]. The examiner places his hand at the patient’s elbow, and with the other hand resists the patient’s extension of his wrist. Pain is elicited at the elbow. A second test is to elicit pain with passive wrist flexion while the elbow is extended [9].

Patients often report a unique discomfort with shaking hands, shaving, lifting luggage or groceries with an extended elbow, or raising a coffee mug. Physical examination on an extended elbow reveals the reproduction of symptoms with resisted middle finger and wrist extension, and examination on a flexed elbow will reproduce pain if the degenerative process is more severe [3]. Imaging studies, such as ultrasound (US) and magnetic resonance imaging, have high sensitivity but lower specificity in detecting LE. Structural abnormalities identified on imaging tend to be consistent across all tendinopathies, and include focal hypoechoic regions, tendon thickening, neovascularization, disruption of fibrils and intrasubstance tears [10].

### 3.3. Provocative Test’s

#### 3.3.1. Maudsley Test

The Maudsley test requires the patient to rest the pronated forearm on a table or examination couch; the patient is then instructed to extend the third digit against resistance, while the examiner palpates the lateral epicondyle. The test is considered positive when palpation reproduces pain over the lateral epicondyle of the humerus. Previous studies evaluating the diagnostic accuracy of Maudsley’s test have reported a sensitivity of 88% and a positive predictive value of 85% [11].

#### 3.3.2. Cozen’s Test

In Cozen’s test, the patient rests the arm with the elbow extended and, with a closed fist, pronates and radially deviates the forearm against examiner resistance. During the maneuver, the examiner stabilizes the affected elbow while palpating the lateral epicondyle. The test is considered positive when pain is elicited at the lateral epicondyle. Previous studies assessing the diagnostic performance of Cozen’s test have reported a high sensitivity of 91% [12].

#### 3.3.3. Chair Test

The Hawk test is an examination in which a patient is positioned standing or sitting behind a chair and attempts to lift it with the hand pronated and the elbow extended. The test is considered positive if this maneuver causes pain at the lateral epicondyle. Previous studies have introduced the Orthopaedic Research Institute Tennis Elbow Testing System (ORI-TETS), which is designed to quantify the force generated during a simulated chair pick-up test. The same study demonstrated a negative correlation between pain and ORI-TETS outcomes, with increased pain associated with decreased strength [13].

### 3.4. Diagnosis Approaches

#### 3.4.1. Ultrasonography

The core pathophysiology of LE is localized to the common extensor origin and the common extensor tendon of the forearm, which inserts onto the lateral humeral epicondyle. Under repeated mechanical stress and cumulative traction, the tendon fibers sustain micro-injuries and repetitive tears. Histological studies demonstrate that the healing response in chronic LE is disrupted, manifesting as mucinous degeneration, and an angiofibroblastic degenerative cycle severely compromises the integrity of the tendon. Diagnosis of lateral epicondylitis is initiated through clinical history and physical examination, but imaging is critical to confirm the underlying pathology and grade severity [14].

USG evaluation focuses on identifying several classic structural signs. These include focal or diffuse hypoechogenicity, localized tendon thickening, intratendinous calcification and bone surface irregularities. Among these, hypoechogenicity of the common extensor origin exhibits the most balanced diagnostic performance, showing moderate sensitivity and high specificity for identifying chronic tendinopathy. Also, structural bone changes on the lateral epicondyle show moderate sensitivity while cortical irregularities, intratendinous calcifications and the presence of neovascularity on Doppler imaging demonstrate exceptionally high specificity [15,16].

Ultrasound scanning requires precise patient positioning, optimal transducer selection and structured scanning sweeps across the lateral elbow compartment to ensure highly reproducible scans and prevent diagnostic errors. The patient is typically seated facing the sonographer. To evaluate the lateral compartment, the elbow must be flexed to 90° and the forearm is fully pronated with the palm resting flat and relaxed on the support table. In alternative configuration, the elbow is flexed to 90° and the forearm is maintained in a relaxed, neutral “thumb-up” position [14,17]. A linear array transducer operating at range 12–17 MHz is recommended [18]. The common extensor tendon is imagined in both longitudinal and transverse planes. The quantify tendon thickness and track therapeutic remodeling examiners use two standardized measurement locations. In the first location the common extensor tendon is measured perpendicular to its long axis at a point exactly 1 cm distal to its insertion on the tip of the lateral epicondyle. In the second, the measurement is made at the flat aspect of the capitellum located between the tendon insertion and the radiohumeral joint space [19]. USG can also assess neovascularization using the Doppler method. Normal tendons are largely avascular at their insertion. However, chronic mechanical overload triggers an angiogenic cascade. To evaluate this, Power Doppler USG is used, which measures the amplitude of the backscattered signal [20].

#### 3.4.2. Magnetic Resonance Imaging

Magnetic resonance imaging (MRI) is essential for detecting the classic structural changes associated with common extensor tendinopathy. Typical findings include focal or diffuse thickening of the tendon, disruption of its normal low-signal fibrillar pattern, and elevated signal intensity on fluid-sensitive sequences. While intratendinous signal changes and partial-thickness tears are highly sensitive markers for chronic tendinopathy, their specificity is sometimes lower than that of clinical examination alone [10]. Beyond tendon itself, MRI provides valuable information on adjacent osseous structures, such as bone marrow edema at the lateral epicondyle, and helps rule out concurrent issues like intra-articular loose bodies or radial collateral ligament tears [7].

Achieving high-quality, reproducible scans of the elbow relies heavily on patient positioning and appropriate sequence selection. Patients are traditionally scanned in the supine position with their arm resting by their side. However, placing the patient prone with the arm extended overhead (the “Superman” position) is often preferred, as it brings the elbow closer to the isocenter of the magnet and significantly improves field homogeneity. The use of a dedicated elbow surface coil at high field strengths (1.5 or 3.0 T) is standard practice. Routine evaluation of the common extensor tendon requires both coronal and axial imaging. A standard protocol includes T1-weighted sequences for anatomical detail, paired with fat-suppressed T2-weighted or proton density (PD) sequences to accurately visualize intrasubstance microtears, peritendinous inflammation, and fluid accumulation [21].

### 3.5. Risk Factors

The development of LE is influenced by multiple risk factors, which can be grouped into four main categories: biomechanical load, individual and systemic predispositions, psychosocial factors, and anatomical or comorbid conditions.

#### 3.5.1. Mechanical Load & Manual Work

Repetitive wrist and forearm movements performed for more than 2 h per day, particularly motions involving extension, pronation, and supination, have been shown to significantly increase the risk of developing lateral epicondylitis, with odds ratios ranging from 2.8 to 4.7 [22]. Additionally, a high Strain Index score greater than 5.1 is associated with a higher likelihood of lateral epicondylitis occurrence, with an odds ratio of 1.75 (95% CI 1.11–2.78) [23]. The risk is further elevated by handling heavy tools weighing over 1 kg or lifting loads exceeding 20 kg at least 10 times per day, especially when vibrating tools are involved, with odds ratios ranging between 2 and 3 [22].

#### 3.5.2. Demographic & Lifestyle Factors

Lateral epicondylitis (LE) most commonly affects individuals between the ages of 35 and 54. Some studies indicate that women may be at greater risk, with reported odds ratios ranging from approximately 1.33 to 2.47 [24]. Tobacco smoking has also been identified as a risk factor, with odds ratios around 1.4 to 1.46, although findings on this association vary across studies [24]. Manual labor is consistently linked to a higher incidence of LE, with odds ratios reported between 2.25 and 2.39 [24]. Additionally, a body mass index (BMI) of 25 or greater appears to be weakly associated with the condition, with an odds ratio of approximately 1.12 [25].

#### 3.5.3. Psychosocial Factors

Low job control and poor social support have been associated with an increased risk of developing lateral epicondylitis, with odds ratios of 2.2 and 1.8, respectively [22]. Additionally, continuous forearm rotation—defined as more than 4 h per day or at least 45% of working time—has also been linked to elevated risk, with an odds ratio of 1.85 [13].

#### 3.5.4. Anatomical & Comorbid Conditions

Coexisting rotator cuff tears have been shown to approximately double the likelihood of developing lateral epicondylitis, with an odds ratio of around 2.77 [26]. Additionally, conditions such as carpal tunnel syndrome are frequently observed alongside LE and are associated with a roughly 1.5-fold increase in risk [26]. Anatomical variations, including narrowing of the arcade of Frohse, may also predispose individuals to nerve entrapment syndromes, which could contribute to or exacerbate symptoms [27]. Some anatomic factors, such as flexibility problems, aging, and poor blood circulation, could favor the development of the injury [28]. Studies using grayscale ultrasonography and color Doppler followed by anesthetic injection show that the development of neovessels in the common extensor origin is associated with pain in lateral epicondylitis [9]. Common methods of release may be performed via percutaneous, arthroscopic or open approaches [3]. Table 1 presents a summary of risk factors associated with LE development.

### 3.6. Treatment Approaches

Although LE tends to resolve spontaneously within one to two years in a substantial proportion of patients, the associated pain and functional impairment often necessitate active intervention. A wide range of treatment strategies has therefore been developed, ranging from conservative measures to surgical approaches, as outlined below [5]. Conservative treatment consists of rest, use of a brace, physical therapy, and use of non-steroidal anti-inflammatory drugs (NSAIDs) [9]. But a prevailing notion in tendinopathy management is to regard exercise and load management as the key element [10]. It is important to distinguish conservative treatment methods.

#### 3.6.1. Orthoses and Kinesio Taping

The literature indicates that various orthotic devices have been evaluated for their efficacy in improving the outcomes of patients with lateral epicondylitis. Studies comparing the utility of various orthotic devices report the efficacy of orthotic devices in outcomes of patients but fail to conclude which orthotic device works better. Hence, further explorations in this field might be necessary [35].

Kinesio tape (KT) has been extensively used for the treatment of musculoskeletal disorders [36].

KT may increase the range of motion without pain and allow the muscle to produce more force [37]. By gate control theory, the constant somatosensory input by cutaneous stretching could potentially close the “gates” to painful input, which prevents pain sensation from traveling to the central nervous system [38]. KT and rest-and-medication treatments were all effective in terms of pain reduction, functional scores and grip strength at the end of the second week, and the only treatment that continued to be effective in the final week was KT [39].

KT is effective in relieving pain, restoring grip strength, and improving functionality in patients with LE undergoing rehabilitation [36].

#### 3.6.2. Non-Steroidal Anti-Inflammatory Drugs (NSAIDs)

Along with basic analgesics, NSAIDs have long been the first line of treatment for tendinitis at all sites, including the lateral elbow. There are numerous kinds of topical, such as diclofenac or naproxen, and oral (i.e., diclofenac gels, solutions) NSAIDs with or without prescription [40]. In the industrialized world, these medications are among the most commonly prescribed. They are also known to be linked to serious morbidity, especially when it comes to deleterious effects on the heart and gastrointestinal tract [41]. A systematic review conducted by Green et al. found that topical NSAIDs are significantly more effective than placebo with respect to pain [weighted main difference = −1.88, (95% CI: −2.54; −1.21)] and participant satisfaction [relative risk 0.39, (95% CI 0.23; 0.66)] in the short term [40]. NSAIDs function by inhibiting the production of prostaglandins by an enzyme known as cyclooxygenase (COX). The body produces prostaglandins, which resemble hormones and are involved in heat, pain, and inflammation. NSAIDs aid in the relief of heat, inflammation, and mild to moderate pain sensations by lowering prostaglandin synthesis. Prostaglandins are produced by the COX-1 and COX-2 enzymes. Prostaglandins, which sustain platelets and shield the stomach lining, are solely produced by COX-1. It also helps to maintain kidney function. Inflammation or injury to the joints results in the production of COX-2. Nonselective inhibitors make up the majority of NSAIDs. This indicates that they suppress COX-1 as well as COX-2. Nonselective NSAIDs may lower protective stomach prostaglandin levels, which could result in stomach ulcers, because they also affect COX-1. A more recent class of NSAIDs, called coxibs, have less of an adverse effect on the stomach because they selectively inhibit COX-2 [40].

#### 3.6.3. Local Injections

Corticosteroid injection is a local therapy in which a dose of a glucocorticoid, usually triamcinolone or methylprednisolone, is administered peritendinously or into the region of the common extensor tendon origin at the LE. The assumed therapeutic actions include the suppression of inflammatory mediators and nociceptive signaling, which caused rapid symptomatic relief [42]. Corticosteroid injection is among the common first-line interventions for LE. Observational analyses have documented that approximately 17–40% of patients with LE receive at least one corticosteroid injection [21,43]. Injection of corticosteroids produces substantial and clinically meaningful short-term pain relief. In a study published in 2013 by Coombes et al., corticosteroid injection alone was associated with large benefits for worst pain (SMD, 1.77 [99% CI, 1.09–2.44]), resting pain (SMD, 0.87 [99% CI, 0.28–1.46]), pain and disability (SMD, 1.81 [99% CI, 1.13–2.48]), and quality of life (SMD, 1.14 [99% CI, 0.53–1.76]) compared to placebo [42]. In a systematic review published in 2010, Coombes et al. confirmed that corticosteroid injection reduced pain to a large extent compared with no intervention in the short term (SMD 1.44, 95% CI 1.17–1.71), but no intervention was favored in the intermediate term (−0.40, −0.67 to −0.14) and long term (−0.31, −0.61 to −0.01) [44]. Collagen injections are currently being used more and frequently in the treatment of LE. Injected collagen acts as a biocompatible type-I collagen scaffold at the site of tendon degeneration, providing a matrix that promotes tenocyte and fibroblast adhesion, migration and proliferation, and supports endogenous collagen synthesis [45]. In a study reported by Farkash et al. in 2019 [46], injectable gel composed of cross-linked bioengineered recombinant human type I collagen combined with autologous platelet-rich plasma was used. Clinical evaluation revealed an improvement in the mean Patient-Rated Tennis Elbow. As a result of treatment, grip strength increased from 28.8 kg at baseline to 36.8 kg at 6 months. Also, improvements in sonographic tendon appearance were noticeable [46]. In pilot study conducted by Corrado et al. in 2019, a series of 5 type I porcine collagen injections at weekly intervals were used. An average reduction of 55–69% of pain and 58–63% of function at 3-month follow-up were observed [47].

Injections of glycosaminoglycan have also been used as a treatment for TE, mainly due to its function in inhibiting clotting factor formation as well as catabolic enzymes in connective tissue. However, their efficiency has been up to debate. Autologous blood injection has been analyzed as a possible treatment option for TE in various randomized control trials. Systematic reviews and meta-analyses have presented various conclusions regarding the clinical efficiency of this treatment modality. Studies in the past have demonstrated that autologous blood injections may trigger an inflammatory reaction around the affected tendon, which promotes tissue healing with both humoral and cellular mediators [48].

#### 3.6.4. Platelet Rich Plasma

Platelet-rich plasma (PRP) is blood plasma that contains a high concentration of platelets. PRP’s concentrated platelets include growth factors, which are required to initiate and accelerate tissue repair and regeneration.

These bioactive proteins accelerate the healing process, encourage the growth of new blood vessels, and start connective tissue mending and repair. The procedure calls for the evacuation of patient blood, centrifugation, and re-injection of plasma into the lateral epicondyle [49].

In a study performed by Peerbooms et al., 100 patients with LE were randomly assigned to the PRP group or the corticosteroid group. The results showed that 49% of patients in the corticosteroid group and 73% of patients in the PRP group were successful. The corticosteroid group showed an initial improvement followed by a decline, whereas the PRP group demonstrated a gradual and sustained improvement over time [50]. Another study compared local steroid injection with PRP 6 months after PRP treatment; patients with LE had a significant improvement in their VAS and MAYO in contrast to those administered the steroid [51].

#### 3.6.5. Autologous Blood Injection

Autologous blood injection (ABI) has demonstrated therapeutic value in managing LE. Similar to PRP, whole blood delivers growth factors and cellular components believed to enhance vascularization and facilitate tissue repair [42]. One proposed mechanism is that ABI triggers a localized pro-inflammatory response, aiding healing in affected tissues. Some studies suggest ABI and PRP yield comparable improvements in pain [52,53,54]. However, research by Raeissadat et al. and Thanasas et al. indicates that PRP may be more effective than ABI in reducing pain at 6–8 weeks post-injection [54,55]. This difference may be attributed to the higher concentration of growth factors in PRP [56]. Additionally, a systematic review by Chou et al. concluded that while ABI outperforms corticosteroids in pain relief, it remains less effective than PRP [57].

#### 3.6.6. Stem Cell

Stem cell therapy represents an experimental approach for chronic lateral epicondylitis in patients unresponsive to conservative treatment. Available evidence is limited to small preliminary studies, and no high-quality randomized controlled trials have been conducted to date. Whether stem cell injections offer any advantage over corticosteroids or PRP in terms of long-term outcomes remains to be established in adequately powered trials. However, high-quality randomized controlled trials are still needed to establish standardized protocols and confirm long-term safety and efficacy.

#### 3.6.7. Orthotics

Counterforce braces offer immediate short-term pain relief and improved grip strength in lateral epicondylitis, outperforming placebo and wrist splints in RCTs. Kroslak et al. showed reduced pain and better arm function up to 26 weeks compared to placebo [58,59,60]. A meta-analysis by Shahabi et al. found a modest short-term pain benefit in younger patients (<45 years), though physiotherapy proved more effective long-term [61].

#### 3.6.8. Surgery

Surgical treatment is normally reserved for patients who have failed after 6–12 months of nonsurgical treatment. Using open, arthroscopic, or percutaneous methods, the diseased tissue within the ECRB and, on occasion, the extensor digitorum communis is debrided. The optimal surgical strategy for treating LE is a topic of debate. Nirschl was the first to report a 97.7% success rate in 82 patients who had open surgical excision and repair of the diseased ECRB, as well as decortication of the underlying bone [62]. In the USA, newly trained orthopedic surgeons performed 92.2% of open surgeries and 7.8% of arthroscopic procedures for LE patients. Debridement alone, debridement with tendon repair, and percutaneous tenotomy accounted for 6.4%, 46.3%, and 47.3% of open treatment, respectively [63]. Surgical treatment is used only in patients who have failed other therapeutic options. Literature data indicate a frequency of use in approximately 2% of patients [64,65].

#### 3.6.9. Open Surgical Technique

The Nirschl technique describes how to remove the angiofibroblastic hyperplasia and liberate the common extensor origin [66]. Thornton et al. described adding suture anchor repair of the ECRB tendon to the lateral epicondyle and reported increased grip and pinch strengths of 110% and 106%, respectively, when compared with the non-operative limb [67]. According to Wilhelm et al., pain can be effectively eliminated by denervation of the painful area [68].

#### 3.6.10. Arthroscopic Treatment

Arthroscopic surgery is a minimally invasive option for patients with chronic lateral epicondylitis (tennis elbow) unresponsive to conservative management. The extensor carpi radialis brevis origin is usually debrided, and inflammatory synovial tissue is removed. Compared to open techniques, arthroscopy enables faster rehabilitation and the direct visualization of intra-articular pathology. Research has indicated positive results, including notable enhancements in function and discomfort, as well as elevated patient satisfaction levels. After arthroscopic debridement, more than 85% of patients had favorable to outstanding outcomes, according to a Solheim et al. [69]. Additionally, it was discovered that arthroscopic treatment produced results that were on par with open release, plus the advantage of a quicker return to activity [70].

A comparison of outcomes between open and arthroscopic TE surgery has been performed. Although the arthroscopic approach showed higher odds of complications and surgical failure, none of these differences reached statistical significance (*p* > 0.05). Moreover, the arthroscopic procedure tended to be longer [71].

#### 3.6.11. Percutaneous Approach

The percutaneous approach involves the minimally invasive release of the common extensor origin through a small skin incision, often under local anesthesia. This technique preserves surrounding tissues, reduces operative time, and facilitates faster recovery. Studies suggest comparable clinical outcomes to open and arthroscopic procedures. Dunkow et al. found no significant difference in pain or function between percutaneous and open techniques, though the former offered quicker return to activity [72]. Similarly, Karkhanis et al. reported that percutaneous release provided equivalent results to arthroscopic surgery at 12-month follow-up [73].

#### 3.6.12. Hyaluronic Acid

Hyaluronic acid injection (HA) is considered a minimally invasive alternative to corticosteroids or surgery, with a favorable safety profile [21,74]. Injection for lateral epicondylitis consists of peritendinous administration of a high- or medium-molecular-weight sodium hyaluronate preparation into the region of the common extensor tendon origin, often with ultrasound guidance [75]. The rationale is based on the viscoelastic, anti-inflammatory, and chondroprotective properties of hyaluronic acid, which may reduce nociceptor stimulation, improve tendon gliding, and support local tissue repair [76]. Clinical protocols vary, but most studies report one to three injections administered at weekly intervals, with follow-up ranging from weeks to one year [77].

HA injections for chronic lateral epicondylitis present an emerging conservative therapy. A randomized trial demonstrated substantial pain reduction (VAS from ~76 to ~14) and functional improvement (QuickDASH, PRTEE) sustained at 12 months in patients with prolonged (>6 months) symptoms, with all treated subjects exceeding minimal clinically important differences [76,78]. A broader network meta-analysis also noted hyaluronic acid (SMD ~−5.6) as more efficacious than placebo injections for lateral epicondylitis, though it included only one HA trial and overall evidence remains limited [77,78]. While head-to-head comparisons with surgical or arthroscopic approaches are lacking, available evidence remains limited and inconsistent, and definitive conclusions regarding the durability of HA injections cannot yet be drawn.

#### 3.6.13. Botulinum Toxin

Injections of botulinum toxin type A (BT-A) into the extensor muscles of the forearm have been studied as a treatment for persistent tennis elbow, particularly in cases when conservative measures are ineffective. Although there are early reports of transitory finger-extension paresis and decreased grip strength, meta-analyses show moderate decreases in pain (effect sizes ~−0.5 to −1.4) after 8–12 weeks when compared to placebo [76,77,78,79,80,81]. Despite somewhat greater surgical success rates, a randomized pilot study comparing BT-A to open surgical release (Hohmann procedure) indicated equivalent “good to excellent” outcomes at one- and two-year follow-up (65–75%) [75]. BT-A appears to offer modest short-to-mid-term pain relief, though these findings should be interpreted with caution given the high risk of bias across the underlying trials.

#### 3.6.14. Laser Treatment

For lateral epicondylitis, low-level laser treatment (LLLT) uses targeted irradiation (such as GaAlAs 830 nm or HeNe 632 nm) to lessen pain and promote tendon repair. In randomized controlled trials, LLLT significantly increased grip strength and visual analogue scale (VAS) pain levels at four weeks compared to placebo; however, by six weeks, the effects tended to plateau [65]. While highlighting protocol heterogeneity and inadequate long-term validation, a systematic review and procedural meta-analysis showed dose-dependent anti-inflammatory and collagen-stimulatory effects [76]. Although there were only slight variations in pain relief between groups, LLLT provided better early grip strength increases when compared to traditional modalities like ultrasonography or braces [82].

#### 3.6.15. Low-Level Laser Therapy (LLLT)

Bjordal et al. concluded that LLLT has an anti-inflammatory effect on lateral elbow tendinopathy (LET) [82]. The anti-inflammatory effect was seen in higher therapeutic dose-ranges than the biomodulatory effect on fibroblast cells and collagen fiber production. Diagnostic ultrasonography of tendinopathies has revealed that partial ruptures and tendon matrix degeneration are underdiagnosed if only physical examinations are made. Consequently, the stimulatory LLLT effect on collagen fiber production should probably be beneficial for tendon repair. Another interesting feature was that LLLT with too high power densities or doses (above 100 mW/cm^2^) seemed to inhibit fibroblast activity and collagen fiber production [82].

#### 3.6.16. High-Intensity Laser Therapy (HILT)

Over the last decade, high-intensity laser therapy (HILT) has gained importance for the treatment of different kinds of sports injuries such as tendon injuries, contusions, and muscle spasms. In their study, Akkurt et al. showed that HILT is an effective and safe treatment for LE over both the short and long term [83]. In different study findings there were significant improvements in the pain VAS scores, upper extremity functions, hand grip strength, and life quality scores in both HILT and LLLT treatment groups [84]. These improvements were found to be more significant in favor of the HILT group in the handgrip strength. Although there was more improvement in the HILT group (59.7%) according to the LILT group (53.5%) in pain VAS (visual analog scale) scores, the change was not statistically significant (*p* > 0.05).

#### 3.6.17. Acupuncture

A systematic review by Trinh et al. evaluated the effectiveness of acupuncture in treating LE. The review included six randomized and quasi-randomized trials. Five of six studies showed that acupuncture significantly reduced pain, particularly in the short term. Compared to control treatments, acupuncture demonstrated superior outcomes in pain relief and functional improvement. The evidence supports acupuncture as a potentially effective short-term treatment option for lateral epicondyle pain [85].

Acupuncture is widely used for analgesia caused by numerous pathologies, including TE. The effectiveness of acupuncture for TE was analyzed in a systematic review and meta-analysis conducted by Zhou. In the study, they concluded that acupuncture appeared to be a superior treatment to drug or blocking therapy [86].

#### 3.6.18. Physiotherapy Treatment

According to the review article, in general, PT (physiotherapy treatment) techniques have a positive effect on the symptoms and resolution of the clinical characteristics of LE [4]. Based on the 19 articles relating to extracorporeal shock waves (ESWT), ultrasound (US), conventional physiotherapy techniques (thermotherapy, electrotherapy, cryotherapy) and therapeutic exercises, the authors of that article gathered the most important conclusions about efficiency and compared the individual types of PT previously described. We present those results in the table below (Table 2).

**Table 2:** Summary of physiotherapeutic techniques for the treatment of tennis elbow along with their effectiveness.

Applied Method of LE Treatment	Type of Technique	Influence on Grip Strength/Functionality of an Elbow	Efficacy (Time of Improvement)	Evidence
Extracorporeal Schock Wave Therapy (ESWT)	A high-energy acoustic wave that penetrates soft tissue and produces micro-vibrations in areas affected by pain or disease [87]	Decrease of pain intensity although the grip strength did not improve significantly	Both the acute and chronic LE patients showed significant improvements in pain intensity after 3 months although 6 months after the intervention, the improvement was greater in the acute LE patients	Supported by several randomized controlled trials (RCTs) and comparative clinical studies. Yalvaç et al. [88] and Kubot et al. [89] compared ESWT with ultrasound therapy and reported greater short-term improvements in pain intensity, grip strength, and elbow functionality after ESWT. Köksal et al. [90] additionally confirmed effectiveness in both acute and chronic lateral epicondylitis patients, with long-term pain reduction observed at 6-month follow-up.
Conventional PT treatment	CPT includes activities like stretching, strengthening, endurance training, balance and coordination exercises [91]	Significant improvements in maximum grip strength and functionality; the pain intensity lower than in ESWT	The pain intensity is much lower than ESWT after one month of the intervention; progressive improvement in all variables was significant at 3, 6 and 12 months after the treatment	Conventional physiotherapy protocols were evaluated in multiple RCTs including Eraslan et al. [92], and Olaussen et al. [93]. These interventions commonly included stretching exercises, TENS, cryotherapy, friction massage, and patient education.
Deep transverse friction massage (DTFM)	A physical therapy technique often used to reduce damage and scarring caused by inflammation; it increases blood flow to the joint, which facilitates healing of the tendon by increasing the supply of oxygen transported to the injury [94]	The results showed that pain intensity, grip strength and functionality improved significantly	6 months after the treatment all groups showed significant improvements in all variables compared to corticosteroid treatment	López-de-Celis et al. [95] evaluated diacutaneous fibrolysis in chronic lateral epicondylalgia and found significant improvements in pain intensity, grip strength, and functionality compared with placebo treatment. Yi et al. [96]
Dynamic wrist extension orthosis	Elbow braces/clasps which have a pad placed distal to the lateral epicondyle to compress very locally at the insertion, and therefore, reduces the forces on the common extensor tendon [94]	The maximum grip strength, pain intensity and functionality were improved progressively after the treatment although strength did not improve significantly	9 months after the end of the intervention the maximum grip strength and functionality were improved	Nishizuka et al. [97] investigated the use of a forearm band combined with stretching exercises and demonstrated significant reductions in pain. Nowotny et al. [94] assessed a dynamic wrist orthosis and observed progressive improvements in pain and upper-limb functionality over 9 months.
Biomechanical Taping BMT	A novel taping technique effective in decreasing lateral elbow pain, increasing handgrip strength; BMT can be applied on painful elbows effecting a better grip among patients with LE [98]	Pain intensity, maximum grip strength and functionality showed significant improvements in all patients	One week after the intervention, the group that received SBMT (Standard Biomechanical Taping) as the first technique obtained better scores in pain intensity	Giray et al. [99] and Eraslan et al. [92] demonstrated that kinesiotaping significantly improved pain intensity and upper-extremity functionality. Zhong et al. (2020) performed a meta-analysis of RCTs and concluded that kinesio taping effectively reduces pain in patients with lateral epicondylitis [36].

Another important thing worth mentioning is the fact that there was a discussion about determining the most effective stretching position for the common extensor carpi radialis, which is the most susceptible to LE. The results showed that, immediately after the treatment, patients treated with DF (diacutaneous fibrolysis and vibration) showed significant improvements across all outcome measures (pain intensity and functionality).

#### 3.6.19. Local Cryotherapy

Local cryotherapy (LC) is a therapy in which a small area of the body is exposed to low temperatures. LC induces a number of physiological reactions such as analgesic effects, neuromuscular effects, and anti-inflammatory and antiedema effects [87]. The benefits to LE of lowering tissue temperature include the ability to reduce the extravasation of blood and protein from new capillaries present in tendinopathy and to reduce the metabolic rate of the tendon, which promotes healing in LE. In an article by Radecka and Lubkowska, published in 2022, they tried to verify the direct effect of a one-time local cryotherapy treatment with the use of cryogenic gas on the clinical symptoms of lateral epicondylitis (pain and pain-free grip) and its effect on the myoelectrical activity of extensor carpi muscles at rest on maximal contraction and isometric contraction during fatigue in male office workers [100]. The authors claimed that the present study assessed the effect of LC on pain perception during the provocation test and PFG (pain-free grip), which is impaired in lateral epicondylitis enthesopathy. They concluded that local cryotherapy soothes the clinical symptoms of male office workers with lateral epicondylitis, causing immediate relief after exposure in the form of pain reduction during resisted wrist extension and increased pain-free grip.

#### 3.6.20. Ultrasound Therapy

Ultrasound therapy (US) is commonly used in the treatment of tendon injuries. US is an electrophysical agent which produces deep heat in tissues. Ultrasonic sound waves, which penetrate through the tissue, enhance local blood flow, stimulate inflammatory mediators, and reduce muscle spasm and pain. The results of the present study showed that US therapy was effective in reducing pain and improving functionality in patients with LE [37]. Another study applied US therapy at a frequency of 1 MHz and intensity of 1.5 W/cm^2^ for 5 min for 10 sessions in which the effects of laser therapy, bracing, and US therapy were compared. The authors demonstrated that all three treatment methods decreased pain at the second week of the treatment according to baseline [82]. In addition, the effects of US therapy and laser therapy continued for 6 weeks after treatment. Although all patients were given strengthening and stretching exercises, they found no significant difference in patient grip strength in the US group.

#### 3.6.21. Iontophoresis with NSAID Administration

Iontophoresis is a type of electrotherapy in which a drug is introduced into tissues by the application of a local electrical current. It is based on the principle that in a given electrical field, positively charged drug ions (cations) are repelled by a positive electrode (anode) and are directed to the cathode (negative electrode). Iontophoresis uses two types of current: direct and alternating. This practice has attracted a great deal of interest in applications related to various musculoskeletal disorders such as LE [101]. This method allows the medication to act locally, especially as NSAID can irritate the gastric mucosa and increase the risk of digestive tract bleeding. Iontophoresis of corticosteroids requires a local tissue concentration that is lower than those achieved in injection but higher than those achieved with oral administration and is, therefore, considered to be both safe and effective. It is probable that the transportation of the drug to the intracellular compartment is increased as a result of the intense arterial vasodilation induced by galvanic current. This means that the active substance reaches a higher concentration than it would if applied orally or parenterally [102].

#### 3.6.22. Radial Shock Wave Therapy (RSWT)

A radial shock wave stimulates a much larger area of tissue than a focused extracorporeal shock wave therapy (ESWT). The effective focal zone of the latter is very small; thus, the area of affected tissue that can be treated is also small. The radial shock wave allows the original site of the disease to be treated (e.g., the lateral epicondylar area), as well as other affected areas. According to the article in the author’s experiment, all three types of pain decreased gradually and comparably between groups (focused shock wave therapy FSWT and radial shock wave therapy RSWT) over the observation period [103]. The reduction in pain did not differentiate the groups significantly.

#### 3.6.23. Myofascial Release (MFR)

Myofascial release (MFR) is the application of a low-load, long-duration stretch to the myofascial complex, intended to restore optimal length, decrease pain, and improve function. MFR generally involves slow, sustained pressure (120–300 s) applied to restricted fascial layers either directly (direct technique MFR) or indirectly (indirect technique MFR). It is being used to treat patients with LE. During direct technique MFR, pressure is applied directly on restricted fascia; practitioners use knuckles, elbow, or other tools to slowly sink into the fascia and apply a few kilograms of force to contact the restricted fascia, apply tension, or stretch the fascia [104].

#### 3.6.24. Dry Needling

Dry needling involves the insertion of thin monofilament needles without injecting into, alongside, or around nerves, muscles, or connective tissues for the management of pain and dysfunction in neuromusculoskeletal conditions. According to the article, deep dry needling was performed, and the needles were rotated three to four times after penetration [105]. Based on the before and after-treatment comparison of the PRTEE (pain and functional) scores, the dry needling group showed significant recovery at both three weeks and six months. Dry needling appeared to outperform first-line conservative measures; however, these results should be regarded as preliminary and require confirmation in larger randomized controlled trials.

#### 3.6.25. Current Trends

According to this systematic review, the main current trend in lateral epicondylitis rehabilitation is a shift from single-modality care to individualized, multimodal treatment, in order to support manual therapy and eccentric strengthening [106]. The review concludes that these two approaches have the most beneficial effects, while shock waves, taping, and orthoses are mainly used as an additional procedure to improve outcomes.

## 4. Limitations

Several limitations of the present review merit acknowledgment. First, the literature search was conducted in a non-systematic manner, which may have introduced selection bias and limits the reproducibility of the search process. Second, no formal quality assessment of included studies was performed, and no evidence grading framework such as GRADE was applied; conclusions therefore reflect a narrative synthesis rather than a hierarchical appraisal of evidence. Third, the included studies vary considerably in design, sample size, follow-up duration, and outcome measures, which precludes direct comparison across treatment modalities. These limitations should be taken into account when interpreting the findings presented herein.

## 5. Conclusions

The first-line treatment of LE is appropriate physiotherapy PT treatment including epicondylar muscle strengthening exercises [5]. Treating lateral epicondylitis can be frustrating for both patients and clinicians [8]. However, 4–11% of patients require surgical intervention, and many patients with severe functional impairment or pain require other nonsurgical treatments [107]. There is an assumption that tennis elbow patients have more depressive feelings and male tennis elbow patients are more positive perfectionists. The diagnosis of tennis elbow is initially clinical in nature. However, in chronic cases, ultrasound, radiographic examination and MRI might be useful to exclude other causes of lateral elbow pain [108]. It is crucial to have a thorough grasp of the long-term effectiveness of specific treatment strategies [108]. LE is a challenging tendinopathic condition with a complex underlying etiology. There is a growing body of evidence that provides some clarity as to what we should and should not be considering in our management of patients with LE [10]. Based on the existing clinical evidence, it was determined that the use of PRP, ABI or surgery is associated with high LE treatment outcomes, which is consistent with the results of Bonczar et al. [109]. Because a long duration does not affect prognosis, it should not be used to justify interventions with questionable efficacy such as surgery [110]. The open approach leads to greater visualization of the operative field and pathologic tissue; however, it is associated with a higher incidence of complications and a longer time to return to work. The arthroscopic approach leads to a shorter recovery, but it is more technically demanding. Current comparative studies have not demonstrated the superiority of any single surgical technique; however, this conclusion is based predominantly on observational data and retrospective series with variable methodology, limited sample sizes, and short follow-up periods. High-quality randomized controlled trials directly comparing open, arthroscopic, and percutaneous approaches remain scarce, and reported success rates should therefore be interpreted within the context of these methodological limitations. Overall, current literature suggests stepwise and individualized treatment strategies for LE. Conserving care should be the first-line treatment for most individuals, based on physiotherapy. Corticosteroid injections, PRP therapy, or even autologous blood therapy may provide some advantages for certain chronic conditions but have shown inconsistent long-term effects. Surgery may be considered in patients with persistent symptoms despite 6–12 months of conservative treatment.

## Figures and Tables

**Table 1:** Summary of risk factors associated with LE development.

Authors	Methodology; Type of Study	Key Findings	Conclusions
Chen et al. 2024 [24]	Meta-analysis conducted by searching PubMed, Embase, and Web of Science in January 2022; data extraction into a predefined worksheet; quality assessment using the QUIPS tool; calculation of pooled effect sizes with 95% confidence intervals using the R package “meta.”	Female sex (OR = 1.33, *p* < 0.05), smoking history (OR = 1.46, *p* < 0.001), manual labor (OR = 2.39, *p* < 0.001), and hypercholesterolemia (OR = 1.67, *p* < 0.05) were identified as significant risk factors for LE development. Also, authors noticed a possible relation between statin treatment for hypercholesterolemia and LE development.	The study concludes that both modifiable (smoking, hypercholesterolemia, manual labor) and non-modifiable (female sex) factors contribute to LE risk, and it highlights the need for further investigation of a potential association with statin use.
Sayampanathan et al. 2020 [25]	Systematic review and meta-analysis: 1032 articles were initially identified through MEDLINE, Scopus, and Web of Science. After applying exclusion criteria, 33 studies were included in the systematic review, of which 15 were incorporated into the meta- analysis. Data were analyzed using Mantel- Haenszel statistics and random-effects models where appropriate	Female sex (OR = 1.29, *p* < 0.001) and current or past tobacco smoking (OR = 1.49, *p* < 0.001) were associated with higher odds of lateral epicondylitis. No significant association was found for BMI ≥ 25 or current vs. past/no smoking status.	Female sex and current or past tobacco smoking were associated with increased risk of lateral epicondylitis, and further research is needed to explore additional risk factors and underlying mechanisms.
Titchener et al. 2013 [29]	Large case-control study using The Health Improvement Network database, including 4998 patients with lateral epicondylitis individually matched to controls by age, sex, and general practice.	Significant risk factors for lateral epicondylitis included rotator cuff pathology (OR = 4.95), De Quervain’s disease (OR = 2.48), carpal tunnel syndrome (OR = 1.50), oral corticosteroid therapy (OR = 1.68), and past smoking history (OR = 1.20). No significant association was found for diabetes, current smoking, trigger finger, rheumatoid arthritis, alcohol intake, or obesity.	Certain musculoskeletal comorbidities, past smoking, and corticosteroid therapy increase the risk of lateral epicondylitis, highlighting the multifactorial nature of its etiology.
Park et al. 2021 [26]	Cross-sectional study of 937 elbows in a rural population. Participants completed questionnaires, physical exams, imaging, and blood tests. Lateral epicondylitis was diagnosed based on pain, tenderness, and pain during wrist dorsiflexion. Multivariable logistic regression assessed associations with demographic, physical, social, comorbid, and serologic factors.	Prevalence of lateral epicondylitis was 26.1%. Significant risk factors included female sex (OR = 2.47), dominant-side involvement (OR = 3.21), manual labor (OR = 2.25), and ipsilateral rotator cuff tear (OR = 2.77; all *p* < 0.001). Author brought up that no metabolic factors were associated.	Female sex, dominant-side involvement, manual labor, and ipsilateral rotator cuff tear increase the risk of lateral epicondylitis, suggesting that overuse factors play a larger role than metabolic factors.
Tajika et al. 2014 [30]	Cross-sectional study of 422 adults (176 men, 246 women) in a Japanese mountain village. Participants completed questionnaires on demographics, dominant hand, occupational workload, recent elbow pain, and lifestyle factors. Lateral epicondylitis was diagnosed based on self- reported symptoms and clinical examination.	Prevalence of lateral epicondylitis was 3.8% (16/422). Most cases were right- handed (15/16) with involvement of left (n = 8) or right (n = 7) elbow; dominant hand was not associated with affected side (*p* = 1.00). Labor heaviness was not associated with elbow pain. Highest prevalence observed in ages 50–59 (9%; 95% CI, 3.8–17.1%) and in unemployed individuals (14%; 95% CI, 1.1–44.1%).	In this study lateral epicondylitis prevalence was low (3.8%) and showed no significant associations with gender, age, BMI, dominant hand, occupational workload, smoking, or alcohol consumption.
Shiri & Viikari-Juntura 2011 [31]	Systematic review analyzed existing studies to evaluate occupational and demographic risk factors associated with lateral epicondylitis.	Lateral epicondylitis affects about 1.0–1.3% of men and 1.1–4.0% of women. Significant risk factors include repetitive wrist movements and forceful gripping. The condition is most common in individuals aged 40–60 years, with women possibly more affected than men. Diagnosis is clinical, based on symptoms and physical examination.	Occupational factors, particularly repetitive wrist movements and forceful gripping, play a key role in lateral epicondylitis, highlighting the importance of ergonomic interventions and preventive strategies in at-risk populations.
Herquelot et al. 2013 [32]	A cohort study was conducted with 3710 workers from a French region, enrolled between 2002 and 2005, and followed up from 2007 to 2010. Occupational health physicians assessed the presence of lateral epicondylitis, while workers self-reported their occupational exposures. Poisson regression models were used to calculate incidence rate ratios (IRRs) for lateral epicondylitis, separately by sex, using multiple imputed data.	The annual incidence rate of lateral epicondylitis was 1.0 per 100 workers among men and 0.9 among women. Workers aged over 45 years had a higher incidence compared to those under 30 years. High physical exertion combined with elbow flexion/extension or extreme wrist bending (>2 h/day) was a significant risk factor for lateral epicondylitis, with an age-adjusted IRR of 3.2 for men and 3.3 for women exposed at both time points.	The study emphasizes the importance of the temporal dimension of occupational exposures in the incidence of lateral epicondylitis. Further research should evaluate the impact of the duration and repetition of occupational exposures on the incidence of lateral epicondylitis.
Otoshi et al. 2015 [33]	Prospective cohort study. The study involved 2000 participants aged 40–79 years from the LOHAS cohort in Japan. Data on chronic hyperglycemia and other health parameters were collected through medical examinations and self-reported questionnaires. The incidence of lateral epicondylitis was determined based on clinical diagnosis and self-reported symptoms. Statistical analysis was performed to assess the association between chronic hyperglycemia and the risk of developing lateral epicondylitis.	The study found a significant association between chronic hyperglycemia and an increased risk of lateral epicondylitis. Participants with chronic hyperglycemia had a higher incidence of lateral epicondylitis compared to those without chronic hyperglycemia. The adjusted hazard ratio for lateral epicondylitis in individuals with chronic hyperglycemia was 1.75 (95% CI: 1.20–2.55).	Chronic hyperglycemia is a significant risk factor for the development of lateral epicondylitis. The findings suggest the importance of managing blood glucose levels to reduce the risk of musculoskeletal disorders such as lateral epicondylitis. Further studies are needed to explore the underlying mechanisms and to confirm these findings in different populations.
Hegmann et al. 2017 [34]	Observational study based on two large prospective occupational cohorts including a total of 1824 workers. Participants completed structured interviews and physical examinations. Baseline data were analyzed to assess associations between a modified cardiovascular disease (CVD) risk score and three outcomes: (1) lateral elbow pain, (2) positive resisted wrist or middle finger extension test, and (3) a combination of symptoms plus at least one positive physical test. Occupational exposures, personal factors, and psychosocial variables were considered as potential confounders. Odds ratios (OR) and 95% confidence intervals (CI) were calculated.	Higher cardiovascular disease (CVD) risk scores were significantly associated with lateral elbow symptoms, positive resisted wrist or middle finger extension tests, and confirmed lateral epicondylitis. Specifically, the odds of having lateral elbow symptoms were 3.81 times higher (95% CI 2.11–6.85), the odds of a positive resisted test were 2.85 times higher (95% CI 1.59–5.12), and the odds of having both symptoms and a positive test were 6.20 times higher (95% CI 2.04–18.82) among participants with higher CVD risk scores.	The findings suggest a potentially modifiable pathogenic mechanism for lateral epicondylitis, highlighting the possibility of CVD risk factor management as a preventive strategy for LE.

## Data Availability

Not applicable.
